# Identifying Sources of Configurality in Three Face Processing Tasks

**DOI:** 10.3389/fpsyg.2012.00456

**Published:** 2012-11-15

**Authors:** Natalie Mestry, Tamaryn Menneer, Michael J. Wenger, Nick Donnelly

**Affiliations:** ^1^Psychology, University of SouthamptonSouthampton, UK; ^2^Department of Psychology, University of OklahomaNorman, OK, USA

**Keywords:** faces, configural processing, general recognition theory, face processing, inversion, perceptual independence

## Abstract

Participants performed three feature-complete face processing tasks involving detection of changes in: (1) feature size and (2) feature identity in successive matching tasks, and (3) feature orientation. In each experiment, information in the top (eyes) and bottom (mouths) parts of faces were manipulated. All tasks were performed with upright and inverted faces. Data were analyzed first using group-based analysis of signal detection measures (sensitivity and bias), and second using analysis of multidimensional measures of sensitivity and bias along with probit regression models in order to draw inferences about independence and separability as defined within general recognition theory (Ashby and Townsend, 1986). The results highlighted different patterns of perceptual and decisional influences across tasks and orientations. There was evidence of orientation specific configural effects (violations of perceptual independence, perceptual seperability and decisional separabilty) in the Feature Orientation Task. For the Feature Identity Task there were orientation specific performance effects and there was evidence of configural effects (violations of decisional separability) in both orientations. Decisional effects are consistent with previous research (Wenger and Ingvalson, 2002, 2003; Richler et al., 2008; Cornes et al., 2011). Crucially, the probit analysis revealed violations of perceptual independence that remain undetected by marginal analysis.

## Introduction

Studies of face perception have used various tasks to explore how faces are processed as wholes or configurations. Amongst the most common exemplars of these are the whole-part (Davidoff and Donnelly, 1990; Tanaka and Farah, 1993) and composite face tasks (Young et al., 1987). In addition, a face specific effect, the Thatcher illusion (Thompson, 1980; Bartlett and Searcy, 1993), is often used to mark the presence of configural processing (Maurer et al., 2002; Donnelly and Hadwin, 2003). The aim of the current research was to investigate three face processing tasks using quantitative methods that address formal definitions of configurality. Two of these tasks are analogues of composite face and Thatcher illusion tasks. The third is a task manipulating size, which is not related specifically to any standard face processing task but does belong to the family of generic manipulations made when comparing faces to probe faces.

Two conclusions are often made in the face literature. First, upright faces are processed holistically or as configurations. By the holistic account, the perception of whole faces occurs automatically and at cost to the perception of face parts (Davidoff and Donnelly, 1990; Tanaka and Farah, 1993). By the configural account, second-order relationships are formed between features (Diamond and Carey, 1986), and inverted faces are processed with effort and in a piecemeal fashion as features.

These conclusions are based on inferences that are grounded in operational definitions: a pattern of data is taken to be an empirical “signature” of configural representation or processing. The logic underlying the use of operational definitions is straight forward but potentially problematic. If configural representation or processing is in force, then a particular empirical regularity (e.g., improved performance in whole relative to part face matching conditions) must be obtained. However, this is sometimes read as implying that if the empirical regularity is obtained then the configural representation or processing must be in force. In the same vein, this would mean that all fast cars are Ferraris; a conclusion that runs counter to the authors’ experience with their Volkswagens. The point to be made here is that the existence of a particular empirical regularity need not require that configural representation or processing is functioning, even if it is consistent with it. To overcome any potential circularity requires very careful experimentation and analysis to ensure all competing accounts are excluded.

One approach is to use theoretically grounded formal definitions of configural representation or processing (e.g., Ashby and Townsend, 1986; Townsend and Nozawa, 1995; O’Toole et al., 2001). Use of these formal definitions allows mapping between tasks and theories in terms of a set of mediating constructs. These mediating constructs allow tests of multiple ways in which data may map to theory, and allow direct comparisons across tasks. The goal of the present study is to subject three face processing tasks to these tests.

The basis for this work is the set of theoretical definitions of configural representation provided by general recognition theory (GRT, Ashby and Townsend, 1986). In order to link these theoretical definitions to data, we adopt an experimental approach that allows us to collect different types of data, and more data than would typically be the case. Specifically, we use a feature-complete identification paradigm (e.g., Townsend et al., 1981; Ashby and Townsend, 1986; Kadlec and Townsend, 1992a; Kadlec and Hicks, 1998). In this paradigm, separate responses are required for each of the features that can vary across all trials. These data can then be analyzed two ways: (1) using group-based (aggregate) analyses of signal detection measures to determine whether there are differences in sensitivity and bias, and whether any differences are consistent across conditions; and (2) analyzing multidimensional measures of sensitivity and bias in order to draw inferences about independence and separability as defined within GRT (Ashby and Townsend, 1986). This second set of analyses allows inferences to be made at the level of the individual, and in terms specified by GRT.

Within a feature-complete identification paradigm, the evidence for configurality comes from the perception of, or responses to, one feature (e.g., eyes) being shown to be dependent on the status of other features (e.g., mouth). Encoded dimensions (e.g., eyes and mouth) may interact perceptually, either at the level of the individual stimulus or across the set of stimuli, and may also interact decisionally, in the generation of a response. Perceptual interactions are characterized via the shape and locations of the distributions of perceptual evidence that arise from each stimulus type (e.g., eyes-upright, mouth-inverted). These perceptual interactions are represented in GRT by violations of perceptual independence (PI) and/or perceptual separability (PS). Decisional interactions are characterized via the shape and location of decision bounds between the distributions, and are represented in GRT as violations of decisional separability (DS).

**Figure 1:**
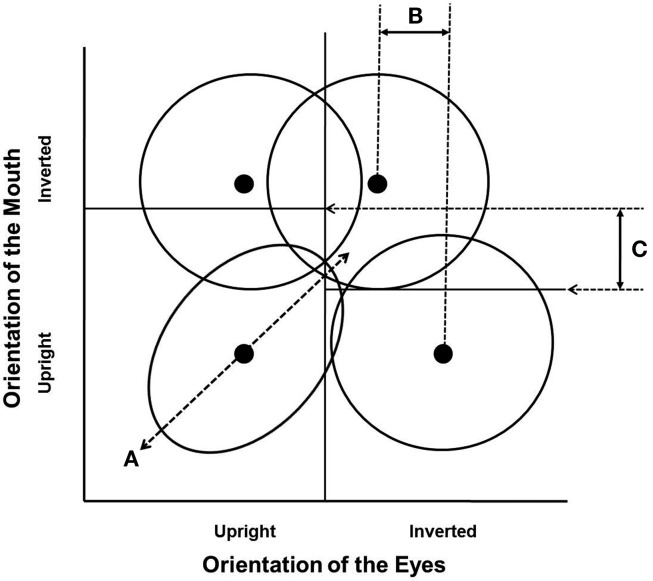
**Schematic representation of three ways of theoretically representing configurality in GRT**. **(A)** illustrates the correlation within the perceptual information predicted by a violation of perceptual independence; **(B)** illustrates the differences in the marginal means for the orientation of the eyes predicted by a violation of perceptual separability; **(C)** illustrates the differences in the location of the decision bounds for the orientation of the mouth as a function of the level of the eyes predicted by a violation of decisional separability.

Figure 1 illustrates violations of perceptual independence, perceptual separability, and decisional separability schematically. Say one dimension of the stimulus is the eyes with levels upright and inverted, and the other dimension is the mouth with levels upright and inverted, then within this two-by-two framework, four stimulus types can be represented by four bivariate probability distributions: one for each combination of the two levels of each dimension. Figure 1 shows these bivariate distributions as four contours (contours of equal likelihood). A violation of perceptual independence in the upright–upright stimulus would be represented by a positive correlation in the bivariate distribution of perceptual information in Figure 1A. This correlation would represent a within-stimulus interaction between the perceptual information for the state of the eyes and mouth. A violation of perceptual separability in Figure 1B would be represented by a shift in the marginal means for one or more levels of each of the two dimensions, such that overall sensitivity to the orientation of the eyes is greater when the mouth is upright than when it is inverted. A violation of decisional separability in Figure 1C would be represented by a shift in the location of the decision bounds that separate the evidence space into response regions, such that when the eyes are upright participants are more likely to respond mouth-upright than when the eyes are inverted. In this way, GRT provides three ways of theoretically defining how features can interact, such that the percept or response to one feature is dependent on the status (or level) of the other feature. GRT therefore provides three ways of theoretically representing configurality.

Formally, a GRT model relies on the parameters of the probability distributions and the extent to which the perceptual evidence supports different responses. In the example just discussed (and represented in Figure 1), if we assume the distributions are bivariate Normal, then each bivariate distribution is completely specified by a vector of means and a covariance matrix:

$$\mu =\left[\begin{array}{c} \mu_{E}\\ \mu_{M} \end{array}\right],\sum_{i}=\left[\begin{array}{cc} \sigma_{E}^{2} & \rho \sigma_{E}\sigma_{M}\\ \rho \sigma_{E}\sigma_{M} & \sigma_{M}^{2} \end{array}\right]$$

If we then assume that the decision bounds are linear, a complete GRT model of hypotheses for configurality is given by four mean vectors and covariance matrices and two or more decision bounds. A violation of perceptual independence for any one stimulus is defined as a non-zero value for the correlation parameter ρ in the covariance matrix for that stimulus. In the example in Figure 1A, ρ for the upright/upright stimulus would be positive. A violation of perceptual separability for one of the stimulus dimensions is defined as a difference in the location parameters (the μs for that dimension in two of the covariance matrices), a difference in the variability parameters (the σs for that dimension in two of the covariance matrices), or both, for one dimension changing across levels of another dimension. In the example in Figure 1B, the marginal mean for eye orientation when the mouth is upright is to the right of the corresponding marginal mean when the mouth is inverted. Finally, a violation of decisional separability is defined as the criterion value(s) for assigning responses in one dimension changing across levels of another dimension. In the example in Figure 1C, the location of the decision bound for the orientation of the mouth when the eyes are upright is above the location of the corresponding decision bound when the eyes are inverted. It is important to realize that any of these violations (or their combinations) could lead to differences in accuracy or RT across the conditions of classic face processing tasks (e.g., recognition across upright versus inverted faces, aligned versus misaligned faces, whole versus part faces) that are used to support inferences about configural processing.

The data obtained in feature-complete factorial designs are typically summarized in an identification/confusion matrix. Two methods of analyzing these data have typically been used in order to draw inferences regarding whether there are any violations of perceptual independence, perceptual separability, or decisional separability. The first of these is the oldest within the tradition of work with GRT. It involves a set of parametric and non-parametric comparisons, and is sometimes referred to collectively as multidimensional signal detection analyses. The second involves one or more methods of directly estimating the parameters of the underlying multivariate Normal distributions. Although these two approaches have not always been used together, they provide two potential sources of converging evidence whose dual use is advantageous given long-standing concerns regarding potential inferential problems (beginning in Ashby and Townsend, 1986).

To date, many of the applications of GRT to questions regarding the perception of, and memory for, faces have reported violations of perceptual separability and decisional separability but rarely violations of perceptual independence (Wenger and Ingvalson, 2002, 2003; Richler et al., 2008; Cornes et al., 2011). On the basis of these results, one might conclude that evidence of configurality in upright faces is driven by shifts in perceptual sensitivity and bias for features in upright relative to inverted faces. This conclusion is at least superficially incongruent with the vernacular conception of configural processing, which speaks to dependencies within a given stimulus (i.e., violation of perceptual independence) as well as relationships between stimuli (i.e., violations of perceptual and decisional separability). This conclusion therefore deserves additional scrutiny.

In the present study we provide the additional scrutiny of this failure to observe violations of perceptual independence with a novel adaptation of a statistical method for estimating the parameters of the underlying multivariate distributions (DeCarlo, 2003). Preliminary work with this approach (Menneer et al., 2009, in preparation) suggests that it may have greater sensitivity to the presence of non-zero correlations than has been true with other methods. The approach uses multiple probit models to directly estimate the parameters of the underlying multivariate Normal distributions (DeCarlo, 2003). By allowing direct estimation of the correlation parameter for each multivariate distribution, there may be a greater chance of detecting violations of perceptual independence in upright faces than has previously been the case. It should be noted that methods used to date have intentionally been conservative with respect to inferring violations of any of the constructs. This method of analysis is different to that used in the previous GRT tasks examining face processing (Wenger and Ingvalson, 2002, 2003; Richler et al., 2008; Cornes et al., 2011), so provides a novel approach to finding evidence for violations of perceptual independence in these tasks. We also compute the multidimensional signal detection measures used in these previous tasks which we refer to as “marginal measures” to examine if there is any consistency in the evidence across the two measures but also with previous evidence outlined by these authors.

In the present study we tested a single group of participants on three, two-alternative forced choice tasks. Our tasks were similar to the three tasks previously investigated, characterized within a GRT framework. Tasks one and two required judgments about successively presented faces: (1) feature size (Wenger and Ingvalson, 2002, 2003), and (2) feature identity (Richler et al., 2008). In the third task participants determined feature orientation (Cornes et al., 2011). The goal was to estimate, for each participant and in each condition, the magnitude of the between-feature, within-stimulus correlations using the probit methods in order to determine whether any evidence exists for the inference of within-stimulus configurality. We also wanted to see whether previous GRT findings would replicate for these three tasks as these three paradigms represent established manipulations to examine face processing. Choosing these tasks, and the stimuli manipulations used, was not to promote them as optimum, but instead to evaluate their suitability for demonstrating perceptual based configural processing at the behavioral level.

## Materials and Methods

### Participants

Seven postgraduate students at the University of Southampton volunteered to take part in the full study in return for payment; four participants were female. Participants had an age range of 22–25 years (*M* = 23.14, *SD* = 1.06). All participants had normal or corrected-to-normal vision. The study was approved by the University of Southampton Ethics Committee and informed consent was obtained from all participants. One participant (number six) was removed from analyses as the probit models were unable converge on a stable solution of GRT parameter estimates, so nothing could be inferred about potential GRT violations for this participant’s data.

### Design

Three tasks were used in this experiment, with all observers performing all three tasks. Each task required participants to judge the status of two features (eyes and mouth, or top and bottom of the face) across two levels (either same versus different or normal orientation versus odd orientation). Together, these two dimensions, each with two levels, created four stimulus conditions. These stimulus conditions were replicated in tasks requiring participants to judge feature size, identity, and orientation. Each task was performed with upright and inverted faces. The set of tasks was then repeated three times by each participant, with a gap of approximately 1 month between repetitions.

In the Feature Size and Feature Identity Tasks, the eyes (top for Feature Identity Task) and the mouth (bottom for Feature Identity Task) were both judged for sameness (yes or no) in a successive matching task. In the Feature Orientation Task, participants judged whether eyes and mouths were the correct orientation relative to the face context (yes or no). For all tasks, judgments about the eyes (top) and the mouth (bottom) were made separately on each trial, but two responses were required on each trial. The order of task was the same across all participants: Feature Size, Identity then Orientation. The order of condition and which feature was responded to first was counterbalanced between participants but remained the same across repeats (with random assignment of possible combinations). The response button was counterbalanced within participant and across repetitions.

### Stimuli

Twenty-five (11 male, 14 female) faces from the NimStim face set (Tottenham et al., 2009) were selected to form a base stimulus set. The faces had no facial hair and blemishes were removed using Adobe Photoshop. Faces were manipulated to equate the positions of the pupil centers and the mouth across the images. Faces were placed within an oval annulus to mask hair and ears. Mean luminance and RMS contrast within the oval were then matched across all stimuli (Adams et al., 2010).

**Figure 2:**
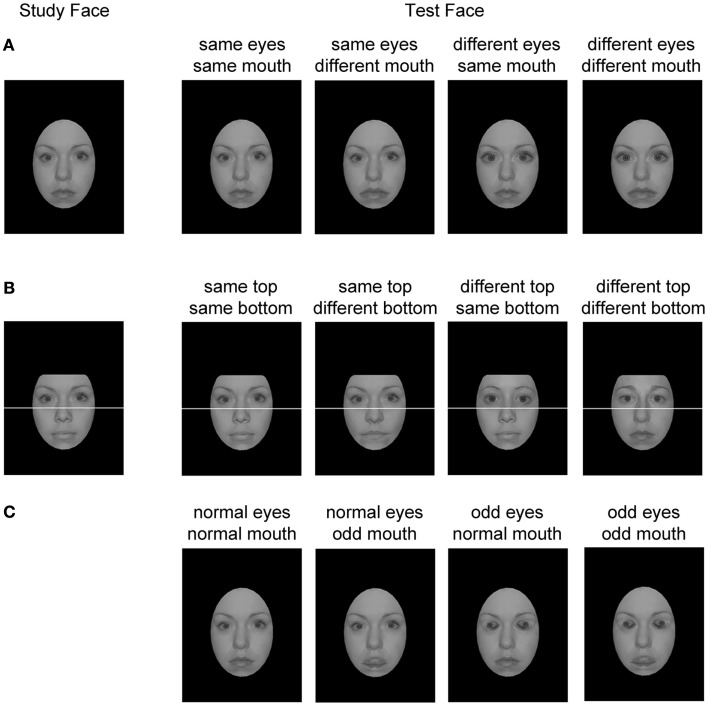
**Examples of stimuli from each of the four conditions in each of the three experiments**. **(A)** Feature Size Task; **(B)** Feature Identity Task; **(C)** Feature Orientation Task.

In the Feature Size Task, 100 gray-scale stimuli were formed from the basic face set. Manipulated features were enlarged by 20% (see Wenger and Ingvalson, 2002, 2003, and Figure 2A for example stimuli). In the Feature Identity Task, composite faces were created from half faces, formed from the original stimulus set divided by a white line (3 pixel diameter) across the bridge of the nose. Only gender-consistent composite faces were formed. Some combinations were rejected due to the failure to make reasonable composites (e.g., bridge of the nose did not line up). After exclusions, 277 composite faces were created which were used to make 100 trial combinations (see Figure 2B). In the Feature Orientation Task, 100 stimuli were created. These consisted of 25 original gray-scale prepared faces, the same 25 faces with inverted (odd) eyes only, inverted (odd) mouths only, or both features inverted (odd). The eyes and mouths in the original stimulus set were manipulated as in Cornes et al. (2011). See Figure 2C for example stimuli.

Faces were presented centrally on the screen at a size of 3.70 cm by 5.00 cm at a viewing distance of 60 cm, creating approximate visual angles of 3.53° and 4.77°. For the Feature Identity Task, this visual angle was 3.53° by 4.10° (6.68° by 4.10° including the white divider line and background) as the top of the forehead was masked to remove the hairline.

In the Feature Size Task, a dot counting task was shown between stimulus and probe faces to reduce the tendency to verbalize responses about size of features in the study face. This was not an issue for the Feature Identity Task as the feature components could not be verbalized in this way and the Feature Orientation Task was classification only (no study face). Dot stimuli in the Feature Size Task were formed from white dots on a black background. The number of dots in the display ranged from one to eight and the size of each individual dot was approximately 0.40 cm diameter. The display of dots subtended visual angles of 6.90° by 8.30° or less in all cases.

The noise mask used throughout the tasks was created using the Gaussian monochromatic noise filter in Adobe Photoshop CS4. It appeared centrally on the screen with a luminance of 15.7 cd/m^2^ [comprised of white (36.20 cd/m^2^) and black (0.11 cd/m^2^) pixels] at a size of 3.70 cm by 5.00 cm, the same visual angle as the stimuli.

### Apparatus and materials

The tasks were built in Experiment Builder (Version 1.5.201). All stimuli were presented against a black background with a screen size of 36.50 cm × 27.50 cm, resolution of 1024 × 768 and refresh rate of 100 Hz. Responses were made via a mouse button press. All text was presented in white. Prompts when responses were required consisted of “yes” and “no” being displayed on the side of the screen corresponding to the correct mouse button, along with a prompt for which question to respond to first. Testing sessions were run in dark room, and observers were seated at a distance of 60 cm from the screen and their head position was maintained using a chin rest.

### Procedure

Participants completed three separate tasks. Three sessions (one for each task) were completed on successive days. Within each session, the upright and inverted conditions of the task were blocked. Each orientation condition of a task contained eight blocks of 54 trials composed of 108 trials of each of the four trial types. The 25 possible stimuli in each trial type were shown at least four times. Ten practice trials were completed before the 432 experimental trials in each orientation condition and the data were not recorded, these also acted as adaptation trials to the dark room. Participants could take short self-paced breaks between blocks. The 3-day experimental cycle was repeated 1 month, then 2 months later giving a total of three repetitions of each task.

All trials were randomized within each session and began with a 500 ms fixation cross requiring participants to look at the center of the screen and ended with a 100 ms noise mask presented after both responses were made. No feedback was given. Only trials where the second response was made within 3 s of the first response were analyzed (see Wenger and Ingvalson, 2002). The three tasks were designed to be similar to the procedures used previous studies (see Experiment 1 of Wenger and Ingvalson, 2002, 2003; Richler et al., 2008; Cornes et al., 2011) while still providing similarities to each other.

#### Feature size task

Participants decided whether both the eyes and mouths of study and test faces were the same size (yes or no). Study faces were presented for 3000 ms. A 100 ms mask and a dot counting task (200 ms display time, with associated response time) were presented between study and test faces. Participants responded either yes on no to a question about the number of dots that had appeared; there were an equal number of yes and no responses. Finally, the test face was displayed and remained visible until both responses were made.

#### Feature identity task

Participants had to decide whether both the top and bottom halves of sequentially presented composite faces were the same (yes or no). Study faces were presented for 400 ms, followed by a 2000 ms mask and then the test face. Test faces remained visible until both responses were made.

#### Feature orientation task

Participants decided if both the eyes and mouth were in the correct orientation relative to the face context (yes or no). If they were then we use the terminology that the features were “normal”. If not, then we describe features as “odd”. Faces were presented for 120 ms and were forward- and backward-masked with a 100 ms noise stimulus.

Note that when comparing the Feature Orientation to the Feature Size and Identity Tasks, “same” trials are being mapped to “normal” orientation and “different” trials are being mapped to “odd” orientation trials. The mapping might seem arbitrary; nevertheless, we reason that finding differences between study and test faces is closer to identifying Thatcherised features than identifying “normal” features. This is because “odd” features in Thatcherised faces, when compared to a mental face norm, would prompt a “different” response. In other words, the “study” face in the Feature Orientation Task is the mental representation of a prototypical face stored in memory.

## Results

Data across the three repetitions were combined for each participant to form six confusion matrices, one for each combination of face orientation and task. These data were analyzed in two ways. First, estimates of sensitivity (*d*′) and bias (*c*) were obtained for each participant in each condition, and in each task (four conditions in each task, representing the two features at two levels). These values were then analyzed across all participants using analysis of variance (ANOVA). The analyses allowed examination of any differences in sensitivity and bias, and whether any differences are consistent across the four stimulus conditions. Specifically we were looking to see if there was any evidence of an interaction of face orientation and status of the other feature (same/different, normal/odd). Such an interaction would be consistent with evidence for whole face processing specific to upright but not inverted faces. Second, marginal and probit analyses were used to characterize potential dependencies between features. These analyses were conducted at the level of the individual observer. Both analyses were used to explore potential violations and to examine the level of agreement across the two methods. Following these analyses, and as a consequence of finding broad agreement across individuals in the patterns of violations, a third analysis was conducted. This third analysis sought to locate the sources of violations of perceptual independence found in the second analysis.

### Group analysis of signal detection measures

Sensitivity (*d*′*)* and bias (*c*) values for discrimination (2AFC paradigm, MacMillan and Creelman, 2005) were calculated for all of the observers in each of the tasks using “same” or “normal” stimuli as the signal distribution. Sensitivity and bias were analyzed in separate 2 (orientation: upright or inverted) × 2 (feature: eyes or mouth) × 2 (status of the other feature: same/normal orientation or different/odd orientation) repeated measures ANOVAs.

#### Feature size task

**Figure 3:**
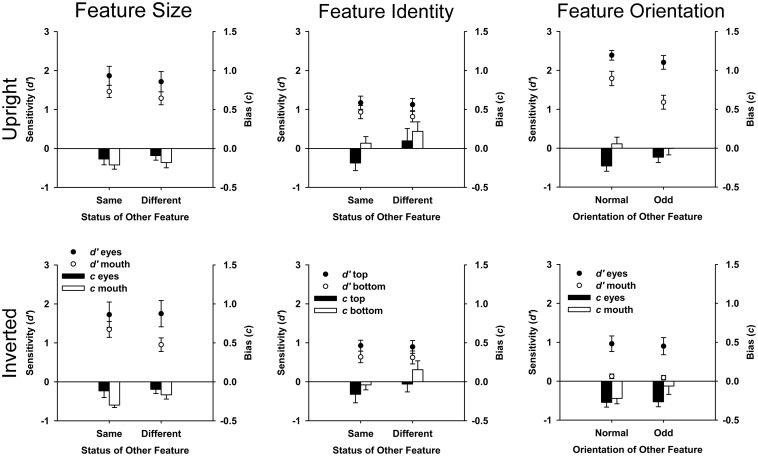
**Plots of sensitivity (*d′*) and bias (*c*) with error bars representing standard error**. Graphs paneled by task (Feature Size, Feature Identity, and Feature Orientation) and orientation condition (upright and inverted). Negative bias values indicate liberal bias to respond “same” (Feature Size and Feature Identity Tasks) or “normal” (Feature Orientation Task). Positive bias values indicate conservative bias, where participants are more likely to respond “different” (Feature Size and Feature Identity Tasks) or “odd” (Feature Orientation Task).

With respect to sensitivity, the main effects of feature and status of the other feature were significant [*F*(1,5) = 8.95, *MSE* = 0.337, *p* = 0.030; *F*(1,5) = 16.36, *MSE* = 0.022, *p* = 0.010]. Sensitivity was higher to the eyes (*M* = 1.76, *SE* = 0.28) than the mouth (*M* = 1.26, *SE* = 0.14). Sensitivity was higher when the other feature was the same (*M* = 1.60, *SE* = 0.21), rather than different (*M* = 1.43, *SE* = 0.21). The main effect of orientation [*F*(1,5) = 0.83, *MSE* = 0.288, *p* = 0.404] failed to reach significance. All interactions were non-significant (all *F*s < 3.31, all *MSE*s** > 0.005, all *p*s > 0.129). With respect to bias, there were no significant main effects or two-way interactions (all *F*s < 4.56, all *MSE*s** > 0.003, all *p*s > 0.593). The three-way interaction of orientation, feature, and status of the other feature was significant [*F*(1,5) = 7.34, *MSE* = 0.002, *p* = 0.042], showing a differential effect of status of the other feature across the eyes and mouth when faces were upright but not when inverted (see Figure 3).

#### Feature identity task

With respect to sensitivity, the main effect of orientation was significant [*F*(1,5) = 24.11, *MSE* = 0.029, *p* = 0.004]. Participants were more sensitive to upright (*M* = 1.01, *SE* = 0.13) than to inverted faces (*M* = 0.77, *SE* = 0.10). No other main effects and, importantly, no interactions reached significance (all *F*s < 4.80, all *MSE*s** > 0.004, all *p*s > 0.08).

With respect to bias, the main effects of orientation, feature, and status of the other feature were significant [*F*(1,5) = 8.83, *MSE* = 0.06, *p* = 0.031; *F*(1,5) = 17.67, *MSE* = 0.020, *p* = 0.008; *F*(1,5) = 22.38, *MSE* = 0.019, *p* = 0.005]. Participants were more likely to respond “same” in the inverted (*M* = −0.02, *SE* = 0.09) than upright (*M* = 0.05, *SE* = 0.11) condition, to the bottom part (*M* = −0.07, *SE* = 0.12) than to the top part (*M* = 0.10, *SE* = 0.09), and when status of the other feature was “different” (*M* = 0.11, *SE* = 0.12) than “same” (*M* = −0.08, *SE* = 0.09). No interactions reached significance (all *F*s < 3.19, all *MSE*s** > 0.003, all *p*s > 0.134, see Figure 3).

#### Feature orientation task

With respect to sensitivity, the main effects of orientation, feature, and status of the other feature were significant [*F*(1,5) = 88.80, *MSE* = 0.255, *p* < 0.001; *F*(1,5) = 31.49, *MSE* = 0.254, *p* = 0.002; *F*(1,5) = 329.78, *MSE* = 0.002, *p* < 0.001]. Sensitivity was higher to upright (*M* = 1.89, *SE* = 0.15) than inverted faces (*M* = 0.52, *SE* = 0.11), to eyes (*M* = 1.61, *SE* = 0.16) than mouths (*M* = 0.80, *SE* = 0.11), and when status of the other feature was “normal” (*M* = 1.32, *SE* = 0.11) than “odd” (*M* = 1.10, *SE* = 0.11). The interaction between orientation and status of the other feature was significant [*F*(1,5) = 35.50, *MSE* = 0.010, *p* = 0.002]. Sensitivity was significantly higher to upright faces [*F*(1,5) = 107.72, *MSE* = 0.009, *p* < 0.001] when the other feature was “normal” (*M* = 2.09, *SE* = 0.15) than when “odd” (*M* = 1.69, *SE* = 0.16), in contrast there was no significant effect of status of the other feature for inverted faces [*F*(1,5) = 4, *MSE* = 0.003, *p* = 0.10]. The three-way interaction was also significant [*F*(1,5) = 14.36, *MSE* = 0.011, *p* = 0.013, see Figure 3] showing a greater effect of status of the other feature for mouths than eyes when faces were upright than inverted. All other interactions were non-significant (all *F*s < 3.41, all *MSE*s** > 0.035, all *p*s > 0.124).

With respect to bias, the main effects of orientation, feature, and status of the other feature were significant [*F*(1,5) = 8.92, *MSE* = 0.024, *p* = 0.031; *F*(1,5) = 13.02, *MSE* = 0.024, *p* = 0.015; *F*(1,5) = 12.18, *MSE* = 0.003, *p* = 0.017]. Participants were less likely to respond “normal” in the upright (*M* = −0.07, *SE* = 0.06) than inverted condition (*M* = −0.20, *SE* = 0.07), to the mouth (*M* = −0.06, *SE* = 0.08) than the eyes (*M* = −0.21, *SE* = 0.05) and when the other feature was “odd” (*M* = −0.11, *SE* = 0.07) than “normal” (*M* = −0.16, *SE* = 0.06). No interactions reached significance (all *F*s < 6.55, all *MSE*s** > 0.004, all *p*s > 0.05; Figure 3).

### Marginal analyses

Multidimensional signal detection analyses combine a set of non-parametric comparisons (Ashby and Townsend, 1986) and comparisons of parametric (typically Normal) measures of sensitivity and bias. They do so for one of the stimulus dimensions across levels of the other stimulus dimensions (Ashby and Townsend, 1986; Kadlec and Townsend, 1992a,b), e.g., sensitivity to eyes across mouth-normal versus mouth-odd. Calculations of the measures of sensitivity and bias are performed in the same way as in one-dimensional signal detection theory (see MacMillan and Creelman, 2005). The results of these comparisons are combined (using the logic in, e.g., Kadlec and Townsend, 1992a,b) to guide inferences regarding potential violations of perceptual independence, perceptual separability, and decisional separability.

Values of *c* and *d*′ provide evidence for inferences about decisional separability and perceptual separability respectively. A non-parametric test of marginal response invariance is also used, in conjunction with the marginal measures of sensitivity and bias, to determine whether decisional separability and perceptual separability hold, using the following equalities.

For *i* = 1, 2:

$$\begin{array}{rcll} & P\left(R_{xiy1}\left|X_{i}Y_{1}\right.\right)+P\left(R_{xiy2}\left|X_{i}Y_{1}\right.\right) & & \\ & \;=P\left(R_{xiy1}\left|X_{i}Y_{2}\right.\right)+P\left(R_{xiy2}\left|X_{i}Y_{2}\right.\right) & & \end{array}$$

For *j* = 1, 2:

$$\begin{array}{rcll} & P\left(R_{x1yj}\left|X_{1}Y_{j}\right.\right)+P\left(R_{x2yj}\left|X_{1}Y_{j}\right.\right) & & \\ & \;=P\left(R_{x1yj}\left|X_{2}Y_{j}\right.\right)+P\left(R_{x2yj}\left|X_{2}Y_{j}\right.\right) & & \end{array}$$

These equalities check whether the probability of responding 1 or 2 (i.e., “same” or “different”, “normal” or “odd”) in the *y*-dimension is the same when *Y* = 1 as it is when *Y* = 2, and similarly check with the probability of responding 1 or 2 in the *x*-dimension is the same regardless of whether *X* = 1 or *X* = 2. If these equalities are satisfied in the data, then tests of equality of *d*′ and *c* can be used to determine if perceptual separability, decisional separability, or both are violated. If the MRI equalities are not satisfied, then inferences regarding perceptual separability and decisional separability become potentially problematic.

Inferences regarding potential violations of perceptual independence are assessed indirectly using a non-parametric test of sampling independence, using the following logic (Ashby and Townsend, 1986; Kadlec and Townsend, 1992a,b). If decisional separability and perceptual independence hold, then the probability of responding *X* = 1 and *Y* = 1, for a given stimulus type (*X_i_Y_j_*), is the joint probability of responding *X* = 1 and of responding *Y* = 1;

$$\begin{array}{rcll} P\left(R_{x1y1}\left|X_{i}Y_{j}\right.\right)= & \left[P\left(R_{x1y1}\left|X_{i}Y_{j}\right.\right)+P\left(R_{x1y2}\left|X_{i}Y_{j}\right.\right)\right] & & \\ & \left[P\left(R_{x1y1}\left|X_{i}Y_{j}\right.\right)+P\left(R_{x2y1}\left|X_{i}Y_{j}\right.\right)\right] & & \end{array}$$

If this equality is not satisfied (the sides of the equation are not equal) in the data for each stimulus type, then this provides evidence for a violation of perceptual independence, contingent on decisional separability holding.

**Figure 4:**
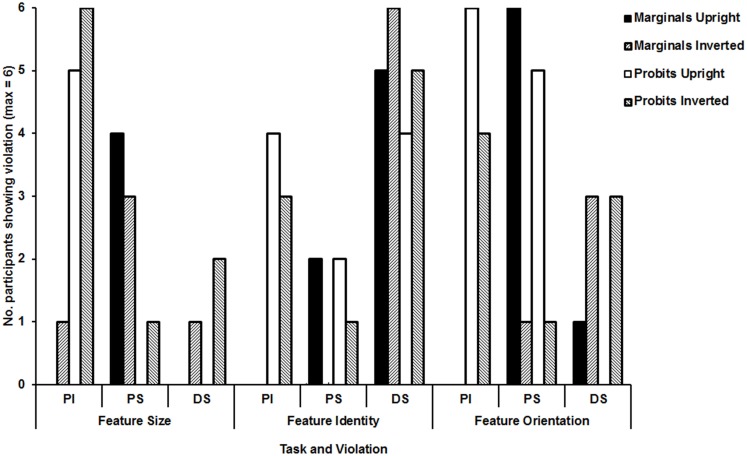
**Summary of significant violations in marginal and probit analyses for each of the tasks and orientation conditions**.

The marginal analyses revealed (1) no violations of perceptual independence in any task (other than for one participant in the inverted condition of the Feature Size Task; see Figure 4); (2) frequent violations of perceptual separability in the upright condition of the Feature Orientation Task with modest numbers of violations in upright and inverted conditions of the Feature Size Task; (3) frequent violations of decisional separability, especially for the Feature Identity Task. All violations of decisional separability were caused by a tendency to give similar responses to both features (i.e., “normal-normal” and “odd-odd” or “same-same” and “different-different”).

### Probit models

The marginal analyses were augmented with direct estimates of parameters of the underlying Normal distributions and the decision bounds. The critical innovation in the present study is the use of probit regression (DeCarlo, 2003) to estimate these quantities. In previous research we have shown probit models to be more sensitive than marginal analyses to the presence of within-stimulus correlations (Menneer et al., 2009, in preparation).

Probit models were implemented using two structures. In the first, each probit model was based on a single distribution (i.e., one for each stimulus), in order to estimate the criteria (*c*s) and the bivariate correlations for each distribution. In the second structure, each probit model was implemented over two neighboring distributions in order to directly estimate *d*′. Both types of model were of the form: y* = β + μ, where y* is the latent dependent variable; β is the regressor for *x*, and provides *d*′; *x* is the explanatory variable (correct response); and μ is the residual distribution, providing bivariate correlations. The outcome *y* depends on the value of y* relative to a criterion, *c*:

$$y=\left\{\begin{array}{c} 0\text{ if }y*\;<\;c\\ 1\text{ if }y*\;\geq \;c \end{array}\right\}$$

In the first structure (one distribution per model), a linear model with a probit link function was implemented for each distribution, in each dimension. The data for each model were restricted to the response data for the given distribution. For each distribution, two models were implemented, one in the *x*-dimension and one in the *y*-dimension, giving eight models in total. Each criterion (*c*) was estimated across two models, one for each of the distributions either side of the criterion. The bivariate correlation for each distribution was estimated from residuals (μ) across two models, one for each dimension within the given distribution.

In the second structure (two distributions per model), a model was implemented for the two distributions in each level of each dimension. For example, for dimension *y* at level 1, there are two distributions: one at *x* = 1 and one at *x* = 2. By including both distributions in the model, the distance between the distribution means (*d*′) can be estimated directly from β. In this way marginal signal detection parameters can be estimated in a two-dimensional case in the same way as specified in DeCarlo (1998) for the one-dimensional case.

In both structures, the criteria were estimated separately for each level within a dimension, hence decisional separability was not enforced (unlike DeCarlo, 2003). Criteria, *d*′s and correlations were estimated separately to avoid under-identification of the models.

There were three key findings: (1) The Feature Identity Task leads to frequent violations of decisional separability in upright and inverted conditions, with violations of decisional separability in the other tasks largely restricted to inverted conditions (see Figure 4); (2) violations of perceptual separability are most commonly found in the upright condition of the Feature Orientation Task; and (3) violations of perceptual independence are reliably present in all tasks and conditions, although somewhat less common in the Feature Identity Task.

### Results summary

The analysis of group means demonstrated that sensitivity was influenced by the status of the other feature (Feature Size and Feature Orientation Tasks), orientation (Feature Identity and Feature Orientation Tasks) and feature (Feature Size and Feature Orientation Tasks). Crucially, the interaction between orientation and status of the other feature was significant in Feature Orientation Task only. In this case, “normal” orientation in one feature enhanced sensitivity to the other feature when faces were upright but not when inverted. The simplest account of this effect is that “normal” orientation allows processing resources to be allocated to regions of potential “inversion”. Seemingly, this allocation of resources can only be realized in upright faces.

With respect to bias, this was influenced by a common series of main effects and interactions in the Feature Identity and Feature Orientation Tasks. These data suggest a generalized effect of orientation, feature type, and status of the other feature on decision-making in response to faces in these tasks.

The analyses motivated by GRT provide further, and different, insights from those available from group means. The marginal and probit analyses converge to suggest: (1) violations of perceptual separability to upright but not inverted faces in the Feature Orientation Task; (2) frequent violations of decisional separability in upright and inverted conditions of the Feature Identity Task; (3) some violations of decisional separability in the inverted conditions of Feature Size and Orientation Tasks found across analyses.

These results confirm an effect of orientation on sensitivity in the Feature Orientation Task that is not present in either of the other tasks. They also confirm that, the Feature Identity Task is influenced by shifts in response criterion across conditions. There is also some evidence that face inversion is associated with a more general tendency to shift criterion across conditions. This latter finding is consistent with face inversion creating a situation where decisions to faces are subject to problem solving strategies.

With respect to violations of perceptual independence, we found evidence for violations of perceptual independence almost exclusively in the probit analyses. The probit analyses found frequent violations of perceptual independence in the Feature Size and Orientation Tasks, in both upright and inverted conditions, with a reduced number in the Feature Identity Task. The marginal analyses suggested only a single violation of perceptual independence. The discrepancy is a cause for concern and might reflect differences in the tendency to make Type I and Type II errors rather than differences in sensitivity.

Our confidence that the difference between probit and marginal analyses reflects sensitivity to violations rather than a tendency to make Type I errors in the case of the probit analysis is strengthened by simulations that we have performed (Menneer et al., 2009, in preparation) in order to compare the two approaches. We created simulated distributions containing known violations of perceptual independence, perceptual separability and decisional separability of various magnitudes, pair-wise combinations of violations, and combinations of all three. A total of 1000 confusion matrices were generated for each violation condition. From each distribution, 250 points were sampled, each of which represents a participant response to a given trial of a given stimulus type. This number is comparable to the 300 trials that were used for each stimulus type in the current experiment. Simulations were created assuming a true *d*′ of 1 or 2 in order to approximate the values found in the current experimental data. Using these data we tested the ability of the probit and marginal models to detect these known violations. The results of these simulations are presented in Table 1.

**Table 1:** **Proportion of 1000 confusion matrices in which violations were detected by marginal and probit analyses in simulated data with known violations**.

Known violation	Analysis:(M)arginals or (P)robits	Violations detected (proportion)					
		
		PI in (1,1)	PI in (2,1)	PI in (1,2)	PI in (2,2)	PS	DS
None	M	0.000	0.000	0.000	0.000	0.028	0.028
	P	0.019	0.016	0.009	0.013	0.002	0.000
PI in (1,1) correlation = −0.25	M	**0.000**	0.000	0.000	0.000	0.025	0.022
	P	0.226	0.023	0.014	0.025	0.001	0.000
PI in (1,1) correlation = +0.25	M	**0.000**	0.000	0.000	0.000	0.024	0.025
	P	0.305	0.024	0.027	0.027	0.002	0.000
PI in (1,1) correlation = −0.5	M	**0.000**	0.000	0.000	0.000	0.031	0.022
	P	0.802	0.016	0.019	0.016	0.002	0.000
PI in (1,1) correlation = +0.5	M	**0.000**	0.000	0.000	0.000	0.024	0.029
	P	0.929	0.013	0.015	0.027	0.002	0.000
PI in all distributions:	M	**0.000**	**0.000**	**0.000**	**0.000**	0.023	0.017
correlation = −0.25	P	0.236	0.301	0.281	0.230	0.002	0.000
PI in all distributions:	M	**0.000**	**0.000**	**0.000**	**0.000**	0.026	0.021
correlation = +0.25	P	0.309	0.208	0.238	0.274	0.004	0.000
PI in all distributions:	M	**0.000**	**0.000**	**0.000**	**0.000**	0.019	0.022
correlation = −0.5	P	0.744	0.938	0.934	0.709	0.001	0.000
PI in all distributions:	M	**0.000**	**0.000**	**0.000**	**0.000**	0.027	0.028
correlation = +0.5	P	0.927	0.726	0.774	0.935	0.002	0.000
PS: *d ′* = 2 versus *d ′* = 2.5	M	0.000	0.000	0.000	0.000	0.578	0.234
	P	0.020	0.014	0.023	0.008	0.347	0.019
PS: *d ′* = 2 versus *d ′* = 3	M	0.000	0.000	0.000	0.000	0.984	0.016
	P	0.015	0.012	0.011	0.030	0.964	0.099
DS with a continuous decision	M	0.000	0.000	0.000	0.000	0.021	0.609
bound: c = 0 versus c = 0.25	P	**0.240**	**0.255**	**0.250**	**0.349**	0.001	0.138
DS with a continuous decision	M	0.000	0.000	0.000	0.000	0.024	0.973
bound: c = 0 versus c = 0.5	P	**0.771**	**0.765**	**0.762**	**0.984**	0.000	0.944
DS with a piecewise decision	M	0.000	0.000	0.000	0.000	0.027	0.621
bound: c = 0 versus c = 0.25	P	0.020	0.014	0.018	0.018	0.000	0.144
DS with a piecewise decision	M	0.000	0.000	0.000	0.000	0.024	0.976
bound: c = 0 versus c = 0.5	P	0.013	0.009	0.009	0.011	0.001	0.942

*Due to space limitations, results are reported for single violations and for *d* ′ = 2 only, although results for *d* ′ = 1 are similar. Inferential errors are shown in bold. Perceptual independence (PI); perceptual separability (PS); decisional separability (DS)*.

For the marginal analyses, results show Type II errors for violations of perceptual independence. For the probit analyses, they show Type I errors for violations of perceptual independence but only when there is a violation of decisional separability with a continuous decision bound (i.e., when the change in the decision bound from one level to the next is continuous, rather than a step function). When this type of violation of decisional separability occurs, correlations in the response confusion matrix can appear as violations of perceptual independence with the same sign and similar magnitude in all distributions. Table 2 contains the mean correlation estimates from the current experimental data. These correlations are not of the same sign within each set of distributions. It is therefore unlikely that the violations of perceptual independence reported in the probit analysis are Type I errors.

**Table 2:** **Mean correlation for each distribution in each task condition with 95% confidence intervals**.

Task	Orientation	Distribution	*M*	*SE*	Lower CI	Upper CI	Different from zero
Feature Size	Inverted	*X*_1_*Y*_1_	0.25	0.13	−0.09	0.58	
		*X*_2_*Y*_1_	−0.40	0.13	−0.71	−0.09	*
		*X*_1_*Y*_2_	−0.26	0.03	−0.33	−0.18	*
		*X*_2_*Y*_2_	0.08	0.09	−0.15	0.31	
	Upright	*X*_1_*Y*_1_	0.34	0.10	0.08	0.60	*
		*X*_2_*Y*_1_	−0.40	0.08	−0.59	−0.21	*
		*X*_1_*Y*_2_	−0.37	0.08	−0.58	−0.16	*
		*X*_2_*Y*_2_	0.08	0.11	−0.20	0.36		
Feature Identity	Inverted	*X*_1_*Y*_1_	0.12	0.07	−0.05	0.29	
		*X*_2_*Y*_1_	−0.02	0.10	−0.26	0.22	
		*X*_1_*Y*_2_	0.07	0.09	−0.15	0.28	
		*X*_2_*Y*_2_	−0.02	0.06	−0.16	0.13	
	Upright	*X*_1_*Y*_1_	0.19	0.08	0.00	0.38	
		*X*_2_*Y*_1_	−0.11	0.12	−0.40	0.18	
		*X*_1_*Y*_2_	−0.09	0.07	−0.28	0.10	
		*X*_2_*Y*_2_	−0.03	0.09	−0.26	0.20		
Feature Orientation	Inverted	*X*_1_*Y*_1_	−0.04	0.09	−0.26	0.17	
		*X*_2_*Y*_1_	−0.10	0.05	−0.23	0.03	
		*X*_1_*Y*_2_	−0.03	0.06	−0.18	0.13	
		*X*_2_*Y*_2_	−0.24	0.06	−0.39	−0.08	*
	Upright	*X*_1_*Y*_1_	0.46	0.10	0.20	0.72	*
		*X*_2_*Y*_1_	−0.41	0.09	−0.63	−0.19	*
		*X*_1_*Y*_2_	−0.43	0.11	−0.70	−0.17	*
		*X*_2_*Y*_2_	−0.07	0.11	−0.35	0.21	

We conclude, therefore, that the probit analyses are more sensitive to violations of perceptual independence than are the marginal analyses. Somewhat troublingly for the vernacular conception of configural processing, the violations of perceptual independence we report are found as frequently in inverted as upright faces. Although these results do support the fact that humans do compute between-feature relationships in faces, they do not support the fact that these computations are done for upright but not inverted faces.

However, there remains one further possibility that violations of perceptual independence are orientation specific. As is standard, we report the existence of a violation of perceptual independence if at least one (out of four) of the bivariate distributions (i.e., stimulus types) exhibits a significant correlation of its underlying dimensions. It is possible that the orientation specificity of violations of perceptual independence exists in differences in the bivariate distributions that show correlations across dimensions.

**Figure 5:**
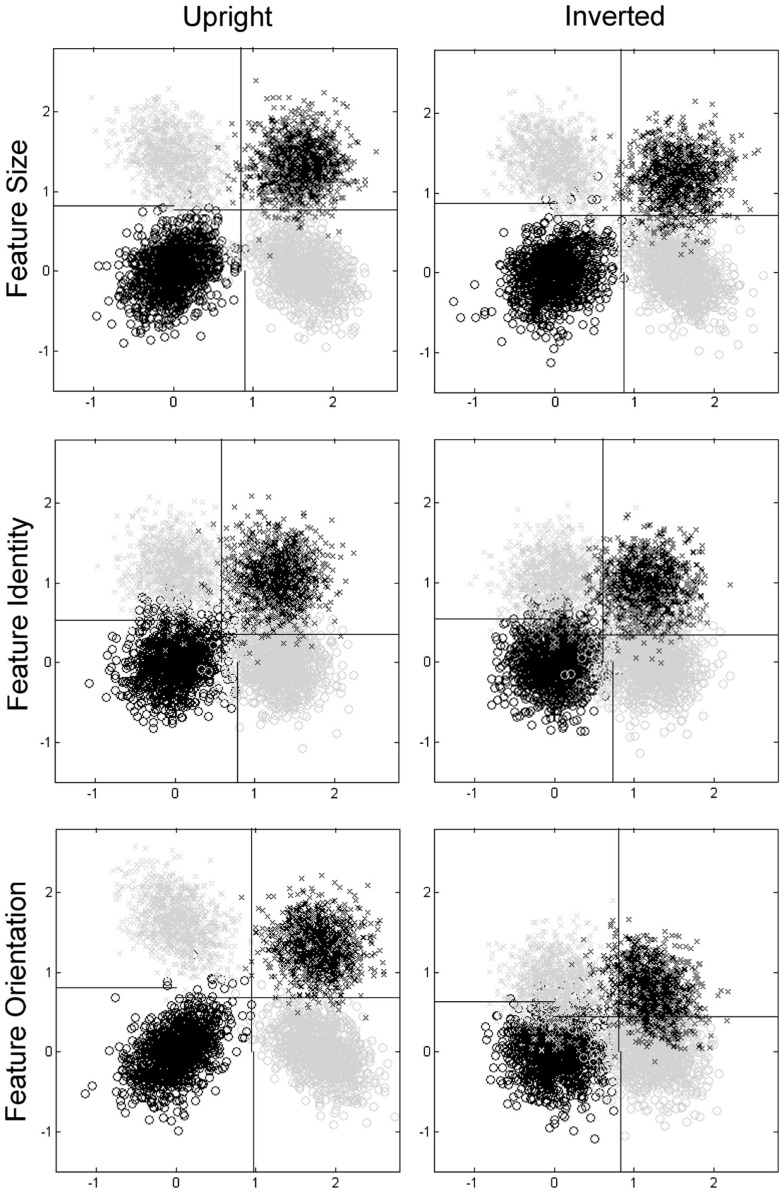
**Plots of distributions and decision criteria from probit analyses**. Same (normal) top, same (normal) bottom = black circles, different (odd) top, same (normal) bottom = gray circles, same (normal) top, different (odd) bottom = gray crosses, different (odd) top, different (odd) bottom = black crosses. Note distributions are plotted relative to an origin at the mean of the bivariate dimensions for the same-same (normal-normal) case. Plots paneled by task (Feature Size, Feature Identity, and Feature Orientation) and orientation condition (upright and inverted).

The mean correlations for bivariate distributions are presented in Table 2. The relative positions of the bivariate distributions and decision criteria in stimulus space for the averaged data are presented in Table 3 for both marginal and probit analyses, and graphically for probit analyses in Figure 5. In these analyses, dimension X is the eye/top feature of the face and dimension Y is the mouth/bottom feature of the face, level 1 is same/normal orientation and level 2 is different/odd orientation. When these data were subjected to ANOVA, the interaction between orientation and distribution was significant in both the Feature Identity Task [*F*(3,15) = 5.14, *MSE* = 0.006, *p* = 0.012] and Feature Orientation Task [*F*(3,15) = 14.03, *MSE* = 0.039, *p* < 0.001] but not the Feature Size Task [*F*(3,15) = 1.16, *MSE* = 0.018, *p* = 0.357].

**Table 3:** **Sensitivity and bias values (and standard errors) for the marginal and probit analyses**.

			Sensitivity (*d*′)				Bias (*c*)			
			
			*X*_1_*Y*_1_ to *X*_2_*Y*_1_	*X*_1_*Y*_1_ to *X*_1_*Y*_2_	*X*_2_*Y*_1_ to *X*_2_*Y*_2_	*X*_1_*Y*_2_ to *X*_2_*Y*_2_	*X*_1_*Y*_1_ to *X*_2_*Y*_1_	*X*_1_*Y*_1_ to *X*_1_*Y*_2_	*X*_2_*Y*_1_ to *X*_2_*Y*_2_	*X*_1_*Y*_2_ to *X*_2_*Y*_2_
Marginals	Inverted	FS	1.72 (0.27)	1.34 (0.14)	0.95 (0.16)	1.75 (0.27)	−0.12 (0.10)	−0.30 (0.05)	−0.17 (0.07)	−0.10 (0.06)
		FI	0.93 (0.11)	0.64 (0.16)	0.62 (0.19)	0.89 (0.14)	−0.16 (0.10)	−0.04 (0.05)	0.15 (0.12)	−0.03 (0.08)
		FO	0.96 (0.24)	0.13 (0.29)	0.09 (0.27)	0.90 (0.23)	−0.27 (0.03)	−0.22 (0.05)	−0.06 (0.07)	−0.27 (0.03)
	Upright	FS	1.87 (0.23)	1.46 (0.08)	1.29 (0.18)	1.71 (0.15)	−0.13 (0.08)	−0.21 (0.04)	−0.18 (0.05)	−0.09 (0.06)
		FI	1.17 (0.14)	0.93 (0.18)	0.82 (0.17)	1.12 (0.14)	−0.19 (0.07)	0.07 (0.07)	0.22 (0.11)	0.10 (0.15)
		FO	2.39 (0.29)	1.79 (0.30)	1.18 (0.22)	2.20 (0.29)	−0.23 (0.06)	0.06 (0.09)	0.00 (0.09)	−0.12 (0.06)
Probits	Inverted	FS	1.58 (0.16)	1.37 (0.08)	1.20 (0.09)	1.56 (0.16)	0.08 (0.06)	0.18 (0.02)	0.12 (0.04)	0.05 (0.03)
		FI	1.21 (0.07)	1.03 (0.07)	0.98 (0.09)	1.16 (0.09)	0.13 (0.09)	0.03 (0.06)	−0.14 (0.11)	0.03 (0.09)
		FO	1.23 (0.08)	0.83 (0.04)	0.77 (0.05)	1.20 (0.09)	0.21 (0.05)	0.22 (0.07)	0.06 (0.11)	0.21 (0.05)
	Upright	FS	1.63 (0.11)	1.41 (0.07)	1.34 (0.08)	1.57 (0.13)	0.08 (0.04)	0.11 (0.02)	0.10 (0.04)	0.06 (0.03)
		FI	1.31 (0.08)	1.16 (0.08)	1.07 (0.07)	1.28 (0.09)	0.13 (0.08)	−0.05 (0.07)	−0.18 (0.11)	−0.06 (0.12)
		FO	1.80 (0.07)	1.64 (0.10)	1.32 (0.08)	1.79 (0.10)	0.07 (0.03)	−0.01 (0.05)	0.02 (0.06)	0.05 (0.03)

**X*_1_*Y*_2_ represents the distribution at level 1 in dimension *X* and level 2 in dimension *Y*. Dimension *X* is the top of the face and dimension *Y* is the bottom of the face, level 1 is same/upright and level 2 is different/odd. FS, Feature Size Task; FI, Feature Identity Task; FO, Feature Orientation Task*.

*Post hoc* analysis of the Feature Orientation and Feature Identity Tasks using Bonferroni corrected paired *t*-tests revealed that, in the Feature Identity Task, there was an effect of orientation for the top-same-bottom-different distribution but not the others [*t*(5) = 8.78, *p* < 0.001], with a positive mean rho value in the inverted condition (*M* = 0.07, *SE* = 0.09) compared to a negative mean rho value in the upright condition (*M* = −0.09, *SE* = 0.07). We are, however, cautious in interpreting this contrast as neither correlation is significantly different from zero.

In the Feature Orientation Task, there was an effect of orientation for the eyes-normal-mouth-normal distribution [*t*(5) = −7.58, *p* = 0.001], with a positive mean rho value in the upright condition (*M* = 0.46, *SE* = 0.10) compared to a negative mean rho value in the inverted condition (*M* = −0.04, *SE* = 0.09). In this case, responses to normal eyes and mouths correlate in upright but not inverted faces. Therefore, these data suggest that violations of perceptual independence are found for typical faces when upright but not when inverted.

This same pattern of results was not found in the other tasks, which may seem surprising but the designs of the experiments differed so a typical, unaltered face was not seen in isolation in either an upright or inverted condition in either the Feature Size or Feature Identity Tasks.

We suggest that these results are important. We are reporting evidence of orientation specific, between-feature encoding but only when feature orientation itself is manipulated.

## General Discussion

The goal of the present study was to explore face processing in three different tasks. Across all tasks we sought evidence of a selective marker of whole face processing in upright faces. In the group-based analyses of *d*′ and *c*, this marker was defined as an interaction between status of the other feature and orientation. We did find evidence of an interaction between status of the other feature and orientation for sensitivity. However, this evidence was only in the Feature Orientation Task where sensitivity was significantly higher to feature orientation in upright faces when the other feature was “normal” than when “odd”. The orientation specific effect found in the group-based analysis for the Feature Orientation Task, was supported by orientation specific violations of perceptual separability and perceptual independence in upright faces, and decisional separability in inverted faces found in the marginal and probit analyses.

The Feature Orientation Task is, of course, an analogue of the Thatcher illusion (Thompson, 1980). The orientation specific violations of perceptual independence that we have are limited to the case where features in their normal orientation are presented. In other words, the orientation specific violations of perceptual independence do not occur with Thatcherised faces (i.e., upright faces with two inverted (odd) features). This finding is in line with the lack of evidence for excitatory interactions between eyes and mouth for the Thatcher illusion, as determined by processing capacity (RT-based) measures (Donnelly et al., 2012). The current finding leaves the orientation specific nature of the Thatcher illusion itself as being driven by violations of perceptual and decisional separability alone. The absence of within-stimulus dependence between eyes and mouth in the Thatcher illusion is at odds with the striking and apparently configural phenomenon experienced when viewing the upright versus inverted stimulus. However, we suggest that the perception of upright Thatcherised faces is facilitated by socio-emotional encoding mechanisms that are activated for upright Thatcherised faces but not inverted (see Donnelly et al., 2011). This additional source of socio-emotional information adds to that available in the visual representation to create the phenomenology associated with the illusion.

The Feature Identity Task is an analogue of the aligned condition of the composite face task. Previous studies of the composite face effect that have also used a feature-complete design also reported a significant role for decisional influences on performance (Richler et al., 2008). Moreover, we note that exploring the aligned condition of the composite face task using the feature-complete design is highly related to use of the so-called “complete design” (as opposed to the partial design: see Richler et al., 2011, for discussion of the differences between partial and complete designs) to explore the impact of alignment and congruency effects on the composite face effect. The “complete design” includes conditions absent from partial designs, where the non-probed feature is manipulated so that, if responses were required for the non-probed feature, correct responses would be either congruent or incongruent with those made to probed feature. Both feature-complete and complete designs index the influence of response congruence. Explicit in the use of both designs is the idea that measuring congruence effects is important in studies of configural and holistic face processing. The present findings confirm that studies of the composite face effect need to include measures of the effect of feature congruence on performance (see also Richler et al., 2011).

The Feature Size Task does not relate specifically to any standard face processing task. Manipulating size does, however, belong to the family of generic manipulations made when comparing faces to probe faces. The data showed no evidence of orientation specific congruency effects.

Across Feature Size, Identity, and Orientation Tasks we need make one general comment. While evidence of orientation specific violations is consistent with a qualitative effect of orientation on face perception, its absence is not. Therefore, only one task (the Feature Orientation Task) demonstrates a qualitative effect. However, it is important to note that orientation may still exert a quantitative influence on performance. Increased sensitivity or bias across orientation is not evidence for dependencies (configurality) between features, but it is important to capture and measure. The enhanced sensitivity to upright over inverted faces in the Feature Size and Identity Tasks indicates just such an effect. Determination of feature size and identity is better for upright than inverted faces, even though, in each task, orientation does not change the fundamental influences on performance. This, of course, connects these effects in face perception to effects of canonical orientation in object identification and perception (e.g., Rock, 1973; Palmer et al., 1981; Tarr and Pinker, 1989).

By using the feature-complete design to explore sources of configurality across a family of related feature manipulations (size, identity, and orientation), it has been possible to begin to directly compare how underlying processes determine differences and similarities across pairs of faces and grotesqueness and typicality within faces. We are at the start of this endeavor and recognize limitations to the conclusions we can currently draw. Our immediate focus has been on establishing statistical tests of sufficient sensitivity, allied to running participants in experimental conditions very similar to those used previously (Wenger and Ingvalson, 2002, 2003; Richler et al., 2008; Cornes et al., 2011). The consequence of this has been that there are increased processing demands for the Feature Size Task, as it included a distractor task, not present in either the Feature Identity or Feature Orientation Tasks. These task differences may have impacted on our differential findings. Also, the nature of asking participants to make decisions about two features on each trial means comparison with previous tasks that only required one response may be limited as the analysis measures necessarily differ.

The current data support the view that the stimulus and task manipulations lead to differences in sources of configurality across tasks. These differences are not readily captured by notions of holistic and second-order relational processing that have been derived for the purpose of explaining face specific processing. Rather these stimulus and task effects are tested against mathematically defined statistical violations, articulated within the general constructs of GRT, that are indicative of dependencies between features. In seeking to establish appropriate tests of these violations, the present study has demonstrated that probit analysis is a useful addition to the set of analytic tools used to draw inferences about configurality within GRT. Furthermore, exploring the correlations between the feature dimensions of bivariate distributions allowed inferences about the stimulus conditions that support the encoding of between-feature, within-face relationships. In doing so, both the presence and magnitude of such relationships can be compared. In sum, using probit analysis allowed report of violations of perceptual independence that remained undetected by marginal analysis.

The present data are consistent with the determination of feature size, identity, and orientation in upright faces being subject to different influences, by demonstrating there are differences between these three tasks. By using a GRT methodology these differences were revealed. If other tasks were explored in this way then the broad similarities and differences between different face processing tasks could be understood. Nevertheless, there were methodological and procedural differences across the tasks reported in the present paper. More effective comparison will require that we now move to test in conditions that are more closely matched across tasks.

## Conflict of Interest Statement

The authors declare that the research was conducted in the absence of any commercial or financial relationships that could be construed as a potential conflict of interest.
